# Salt intake per dish in the Japanese diet: a clue to help establish dietary goals at home

**DOI:** 10.1017/jns.2021.88

**Published:** 2021-12-15

**Authors:** Miyuki Imamoto, Toshihiko Takada, Sho Sasaki, Kenji Kato, Yoshihiro Onishi

**Affiliations:** 1 Department of Food and Human Nutrition, Faculty of Human Life Science, Notre Dame Seishin University, 2-16-9 Ifuku-cho, Kita-ku, Okayama 700-8516, Japan; 2 Research Association for Applied Dietary and Physical Therapy (ADAPT), Ashiya, Japan; 3 Department of General Medicine, Shirakawa Satellite for Teaching And Research (STAR), Fukushima Medical University, Fukushima, Japan; 4 Department of Healthcare Epidemiology, School of Public Health in the Graduate School of Medicine, Kyoto University, Kyoto, Japan; 5 Department of Nephrology/Clinical Research Support Office, Iizuka Hospital, Iizuka, Japan; 6 Center for Innovative Research for Communities and Clinical Excellence (CiRC2LE), Fukushima Medical University, Fukushima, Japan

**Keywords:** Salt reductions, Nutritional balance, Dietary records, Community-dwelling individuals, Japanese diet, CrS, creatinine concentration in spot urine (mg/L), DUSE, daily urinary salt excretion, EDSI, estimated daily salt intake, FBDG, The Food-based Dietary Guideline, NaS, sodium concentration in spot urine (mEq/L), Pr.UCr24, estimated 24-h urinary creatinine excretion, SI_meal_, salt intake per meal, SI_dish_, salt intake per dish

## Abstract

The salt intake of Japanese at home remains high. To aid in salt reduction and encourage a balanced diet, we conducted a cross-sectional study using data from a previous clinical trial in community-dwelling individuals to evaluate major salt sources and relationships among the intake of different dishes in the Japanese diet at home. Dietary records and urinary salt excretion measurements were performed daily for 1 month in seventy-nine participants. The records were classified into six grain dish categories as staple dishes, seven side dish categories and a snack category. Regression analyses were used to estimate (1) salt intake per meal for each category of grain dish, (2) salt intake per dish from each dish category and its contribution to the total salt intake and (3) the influence of grain dish selection on the frequencies of other dishes. Salt intake per meal was approximately 3 g, regardless of grain dish selection. Fish and meat dishes showed the largest contribution to the total salt intake (35 %), followed by vegetable dishes (19 %). The intake of fish or meat and vegetables was promoted by plain rice and reduced by ramen noodles. The intake of dairy products was only promoted by bread, while that of fruits was not influenced by any grain dish category. These results suggest simple strategies to reduce salt intake while maintaining dietary balance, such as eating plain rice more often and using less salt to cook meat/fish and vegetable dishes.

## Graphical abstract

**Figure:**
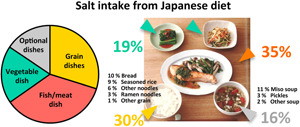

## Introduction

Excessive salt intake has been shown to be the main cause of hypertension, and salt reduction efforts have been implemented worldwide^(1−3)^. In Japan, where both salt intake^(1)^ and the prevalence of hypertension are high^(4)^, reductions in salt intake have stalled since 2014 and remain high at 10⋅9 g/d for men and 9⋅3 g/d for women as of 2019^(5)^, although integrated approaches, including political, social, demographic and individual approaches^(6,7)^, have been adopted. Reinforcing salt reduction at home remains important for further reducing salt intake, as the household diet accounts for up to 70 % of the salt intake in Japan^(8,9)^.

Major sources of salt in the Japanese diet include seasonings, such as soy sauce, and salty foods, such as miso soup, Japanese pickles and salted fish^(10)^, as well as grain dishes with a high salt content, such as ramen noodles and rice bowls^(6)^. As such, current guides on how to reduce salt intake at home^(6)^ include tips such as warning against the consumption of high-salt foods, along with examples of low-salt dishes to prepare and lists of low-salt food products. However, the content of such guides was not found to be quantitatively linked to salt intake in the daily diet, thus limiting their ability to help set a comprehensive goal (i.e. what and how much to eat) for salt reduction.

A distinctive feature of the Japanese diet is that each meal comprises several small dishes combined around a grain dish as staple food, resulting in good nutritional balance^(11)^. Therefore, dietary guidance in Japan might be more effective if based on dishes^(12)^ rather than on nutrients, foods or dietary patterns, as has been the focus of general nutrition guidance^(13)^. One useful example is the Japanese version of the food-based dietary guideline (FBDG)^(14,15)^ developed as part of a global effort to improve nutritional balance in the diet. The Japanese FBDG uses a highly simplified classification of dishes to make the guideline easier to understand, even for individuals with no interest in nutrition. Similarly, a comprehensive guideline for salt reduction at home may be able to be developed if the salt intake target can be translated into a dietary pattern as a combination of dishes.

To this end, information on the amount of salt in each dish, including salt entering the dish through cooking, needs to be gathered. Therefore, in the present study, we analysed the dietary records and urinary salt excretion of community-dwelling Japanese people collected in a recent clinical trial^(16)^ and estimated the salt intake by dish in order to determine which dietary patterns reduce salt intake while maintaining balance in the Japanese diet.

## Methods

### Study design and participants

We conducted the present study as a secondary analysis of a previous clinical trial^(16)^ in Japan. The clinical trial originally examined the effects of self-monitoring one's salt intake on salt reduction outcomes among healthy individuals ≥20 years old belonging to five communities/workplace in Fukushima, a rural area of Japan. The intervention group self-monitored their urinary salt excretion daily, while the control group did not. All participants recorded their meals every day for 1 month.

The present study involved seventy-nine participants from the intervention group for whom urinary salt excretion was measured. Among these participants, sixty whose dietary records of three meals a day (including skipped meals) and daily salt excretion values were both available for at least 15 d during the 1-month study period were analysed. The participant flowchart is shown in Supplementary Fig. S1.

### Data collection and processing

#### Dietary records

We collected and analysed dietary records based on two ideas. First, we used dishes – rather than nutrients or foods – as meal components to analyse repeated dietary records and intelligible deliverables. Second, we placed importance on grain dishes. A variety of grain dishes are staples in the Japanese diet, including rice, bread, noodles and wheat flour. The selection of a grain dish markedly influences the composition of a given meal. For example, plain rice is normally accompanied by several optional dishes, while bread is rarely combined with miso soup. Therefore, we classified grain dishes into subcategories and regarded them as the major determinants of other dishes.

Participants wrote the name of each dish they ate for breakfast, lunch, dinner and snacks on recording sheets in free-text style (Supplementary Fig. S2). We did not include portion-size measurements, which would have precluded repeated dietary recordings.

The Japanese version of the FBDG^(12)^ classifies meals into the following five dish categories: grains, fish/meat, vegetables, dairy products and fruits. Based on this categorisation, we further classified grain dishes into six subcategories. After adding several high-salt dishes and snacks, we used the dish categories shown in Table 1, which Japanese people unmistakably recognise. Supplementary Table S1 shows examples of actual dish names by categories. Data were processed by a team of six individuals, including an experienced registered dietician, to make the classification consistent. The intakes of dishes were converted to yes/no per meal by each dish category. If a meal contained no grain, it was included in the category ‘meal with no grain’. These data per meal were then summed to assess the frequency of eating each dish per day.

**Table 1.** Dish-level categories of food used in the present study and intake frequency of each category

Name of dish category	Main food items (example)	Frequency (times/week)
**Grain dishes (as staple dishes)**		
Plain rice	Cooked rice without salt	9⋅8 (5⋅9–12⋅4)
Seasoned rice	Rice dishes other than plain rice	3⋅8 (2⋅5–5⋅3)
Bread	Bread-based dishes	3⋅1 (1⋅5–4⋅6)
Ramen noodle	Ramen noodle	0⋅6 (0⋅3–1⋅3)
Other noodles	Noodles other than ramen, including Udon, Soba, pasta, etc.	1⋅5 (1⋅0–2⋅4)
Other grains	Grain dishes other than the above, including Mochi, Japanese pizza	0⋅6 (0⋅3–1⋅0)
(Meal with no grain)		1⋅2 (0⋅3–3⋅2)
**Other dishes**		
Fish/meat dish	Dishes mainly consisting of meat, fish, soybeans and egg	13⋅4 (11⋅3–15⋅1)
Vegetable dish	Dishes mainly consisting of vegetables, mushrooms and seaweed	11⋅8 (8⋅9–15⋅4)
Dairy products	Milk, yogurt, cheese, etc.	3⋅0 (0⋅8–6⋅5)
Fruits	Fruits and canned fruits	4⋅8 (1⋅3–7⋅8)
**Optional dishes**		
Miso soup	Miso soup	4⋅3 (2⋅2–6⋅7)
Other soup	Soups other than miso soup. Corn soup, potage, etc.	0⋅8 (0⋅3–1⋅8)
Pickles	Vegetable or seafood pickled or boiled in soy sauce	2⋅3 (0⋅7–3⋅8)
Snack	Sweets, etc., excluding dairy products and fruits	4⋅3 (2⋅2–8⋅3)

Values are presented as the median (interquartile range).

#### Salt intake

Participants used a self-monitoring device (Salt Monitor KME-03: Kohno ME Laboratory, Kawasaki, Japan)^(17)^ to estimate their daily urinary salt excretion (DUSE) from an overnight urine sample. The device consists of a 1-litre urine cup and an electronic device with volume and conductivity sensors. The volume sensor measures the urine volume in the cup, while the conductivity sensor measures the electric conductivity. The device estimates the 24-h salt excretion assuming an 8-h sleep period, which has been shown to approximate the value measured by the 24-h urine collection method^(18)^ and to detect daily changes in salt intake^(19)^. Participants recorded their DUSE every day during the study period^(16)^. The DUSE was regarded as an estimate of the daily salt intake (EDSI) in the previous day.

Urinary salt excretion was also assessed by spot urine at the beginning of the study using the equation recommended by the Japanese Society of Hypertension^(20,21)^: 24-h salt excretion (g/d) = 0⋅0585 × 21⋅98 × [(NaS/CrS) × Pr.UCr24] × 0⋅392, where NaS is the sodium concentration in spot urine (mEq/L), CrS is the creatinine concentration in spot urine (mg/L) and Pr.UCr24 is the estimated 24-h urinary creatinine excretion (mg/d), with Pr.UCr24 = −2⋅04 × age + 14⋅89 × body weight (kg) + 16⋅14 × height (cm) − 2244⋅45.

### Data analyses

#### Grain dish selection and salt intake

To assess the influence of grain dish selection on salt intake, we used a regression model with EDSI, as follows:1
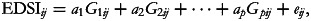
where *i* and *j* respectively represent a subject and a day; *G*_1_, *G*_2_, …, *G_p_* are the frequencies (0, 1, 2 or 3) of selecting each grain category of 1, 2, …, *p* in a day; *a*_1_, *a*_2_, …, *a_p_* are regression coefficients representing the estimated salt intake per meal (SI_meal_) resulting from the grain dish selection and *e* represents an error term. Since the EDSI must be 0 when the frequency of meals is 0, we decided to use a regression model without the intercept term (regression through the origin)^(22,23)^. We used robust variance estimation to deal with within-subject as well as within-household correlations in repeated measurements.

#### Salt intake from each dish category

To identify major sources of salt intake, we used a similar regression model, as follows:2
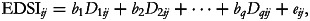
where *D*_1_, *D*_2_, …, *D_q_* are the frequencies (0, 1, 2 or 3) of selecting each dish category of 1, 2, …, *q* in a day; and *b*_1_, *b*_2_, …, *b_q_* are regression coefficients representing the estimated daily salt intake from the dish category (SI_dish_). Plain rice, dairy products and fruits were not included in the model because they contain little or no salt. Snacks were also excluded because the SI_dish_ value was almost 0 in a preliminary regression analysis.

The average contribution of each dish category to the total salt intake was estimated using Eq. 3, as follows:3
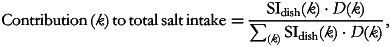
where (*k*) indicates the dish category and *D* is the frequency of its consumption.

#### Association of grain dish selection with other dishes

To examine the association of grain dish selection with the frequency of other dishes, we performed four separate regression analyses with fish/meat dish, vegetable dish, dairy products or fruits as an outcome variable, and each category of grain dishes as explanatory variables. We selected a fixed effect model^(24)^, or within-subject comparison, to remove the influence of individuals’ preferences. In this model, the regression coefficient represents an estimate of an increase/decrease in the frequency of each optional dish in a day caused by a one-dish increment in each grain dish.

### Statistical analyses

Continuous and categorical variables are summarised as medians/interquartile ranges and proportions, respectively. The results of regression analyses are shown as point estimates and 95 % confidence intervals. Stata 14 was used for analyses.

## Results

### Study participants

Table 2 summarises the participant characteristics. The mean age was 62 years, and 75 % of participants were female. Approximately 50 % of participants had been diagnosed with hypertension. The mean salt intake based on spot urine was 9⋅2 g/d. The frequency of eating each dish category is shown in Table 1. Grain dishes were consumed 18⋅6 times per week (89 % in 21 meals). Approximately half of these dishes were plain rice, 1/6 seasoned rice, 1/6 bread and 1/6 other noodles or grains. The frequencies of fish/meat dishes and vegetable dishes were 13⋅2 (63 %) and 11⋅8 (57 %) times, respectively.

**Table 2.** Characteristics of subjects included in the analyses

Number of subjects	60
Observed person-days	1349
Age (years)	60 (50–74)
Sex, F/M	46/14
Body mass index (kg/m^2^)	24⋅0 (20⋅9–27⋅0)
Hypertension	32 (52 %)
Chronic kidney diseases	4 (6 %)
Urinary salt excretion (g/d)	9⋅2 (7⋅1–11⋅4)
Systolic blood pressure (mmHg)	138 (117–159)
Diastolic blood pressure (mmHg)	78 (67–90)

Values are presented as the number (proportion) or median (interquartile range).

### Grain dish selection and salt intake per meal

**Fig. 1.**
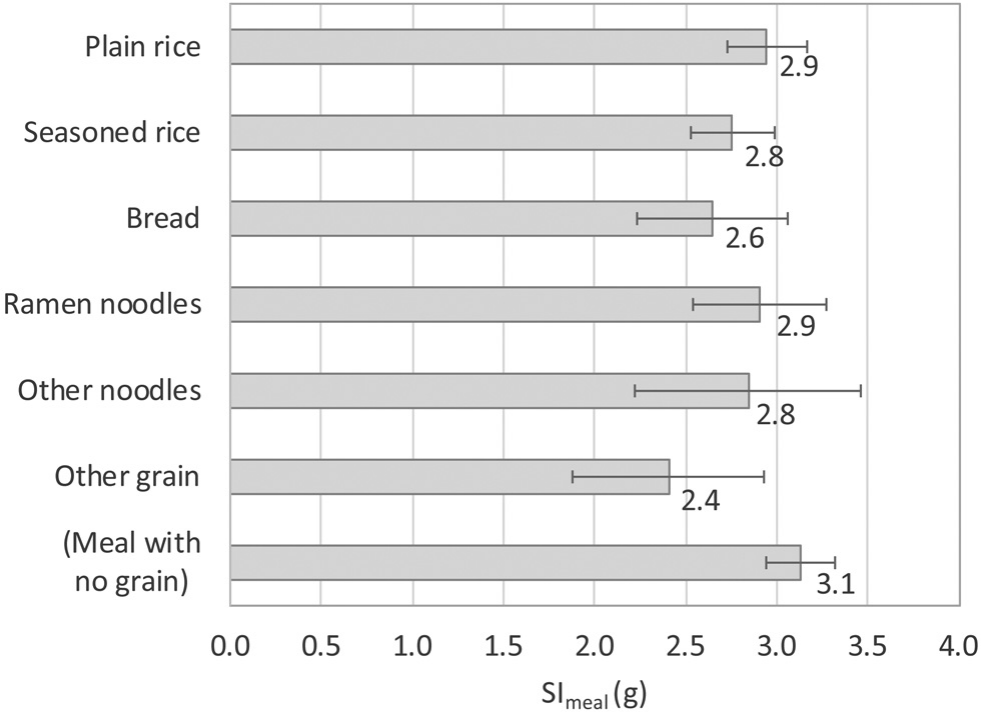
Estimated salt intake per meal (SI_meal_) for each category of grain dishes. The values beside each bar denote point estimates, and the error bars indicate the 95 % confidence intervals.

Fig. 1 shows the SI_meal_ for each grain dish category. Values were similar for all grain dishes, excluding other grains: the SI_meal_ was approximately 3 g and did not vary markedly, even when meals contained no grain dish. Since the frequency of other grains was as low as 0⋅6 times/week, no grain dish selection actually resulted in any marked differences in the salt intake.

### Salt intake from each dish category

**Fig. 2.**
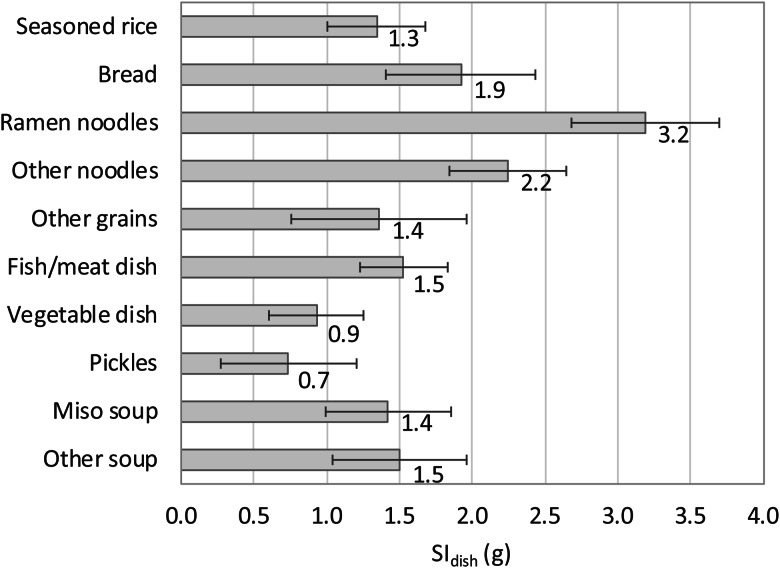
Estimated salt intake per dish (SI_dish_) for each category of dishes. The values beside each bar denote point estimates, and the error bars indicate the 95 % confidence intervals.

**Fig. 3.**
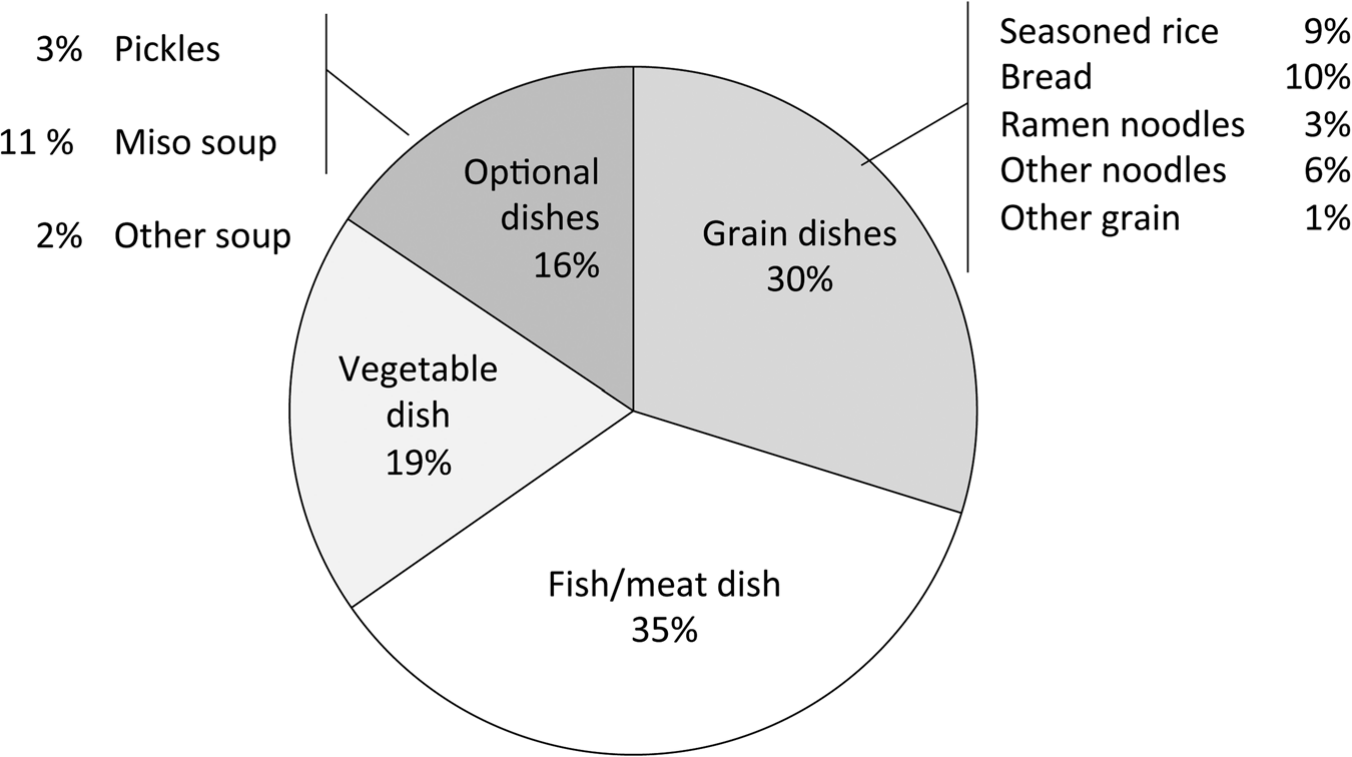
Contribution of each dish category to the total salt intake in the study participants.

Fig. 2 shows the SI_dish_ for each dish category. The estimate was the largest for ramen noodles (3⋅2 g), followed by other noodles (2⋅2 g) and bread (1⋅9 g). Fig. 3 shows the contribution of each dish category to the total salt intake. This contribution was the largest for fish/meat dishes, followed by vegetable dishes; these two categories accounted for more than 50 % of all categories. The value for soups and pickles was smaller than for other dish categories. Among grain dishes, the values for bread and seasoned rice were quite large.

### Association of grain dish selection with the intake of other dishes

**Fig. 4.**
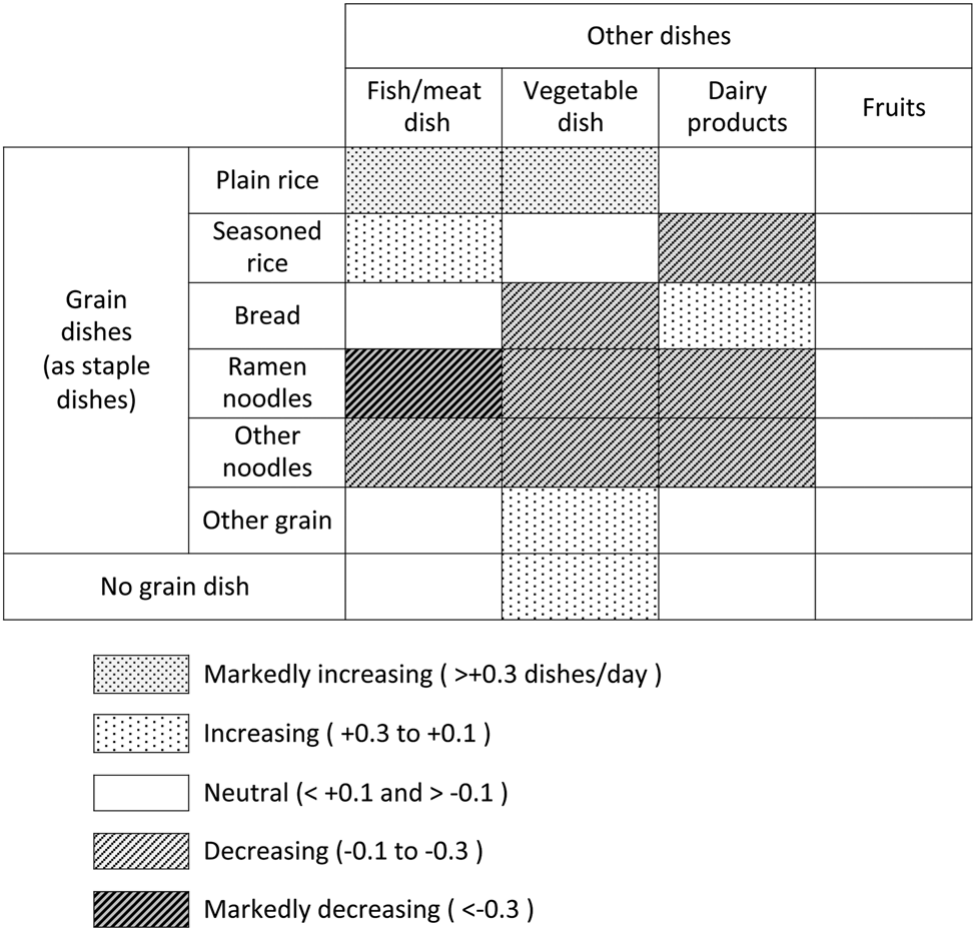
Effects of the intake frequency of each grain dish on that of other dishes.

Fig. 4 shows the association of grain dish selection with the intake of other dishes. The intake of fish/meat and vegetables, which is essential for a balanced meal, was most markedly promoted by plain rice (0⋅31 and 0⋅43 dishes per day, respectively) and reduced the most by ramen noodles (−0⋅48 and −0⋅22). Another promoter of fish/meat intake was seasoned rice (0⋅18). Other promoters of vegetable intake were other grains (0⋅16) and no grains (0⋅18). Other noodles were also associated with reductions in both cases (−0⋅22 and −0⋅22, respectively). The intake of dairy products was promoted by bread (0⋅11). No grain dish promoted fruit intake.

## Discussion

We estimated the average salt intake for each dish and the association between grain dish choices and other dishes in the daily diet of a sample of Japanese community residents. We found that (1) fish/meat and vegetable dishes accounted for more than half of the total salt intake; (2) the amount of salt per meal was approximately 3 g, regardless of the choice of grain dish and (3) the choice of grain dish affected the composition of other dishes. To our knowledge, this is the first attempt to clarify the diet-wide, per-dish intake of salt in the Japanese diet.

A few studies have reported the salt content of a limited number of Japanese dishes. The known salt content per dish (consistent with the categories used in the present study) was 1⋅5 g for miso soup and 5⋅5 g for ramen noodle^(6)^. Two studies reported the contribution of dish categories to the total salt content, with the INTERMAP^(25)^ estimating a value of 10 % for miso soup and 10 % for Japanese pickles and Asakura *et al.*^(9)^ a value of 3–4 % for Japanese pickles and 4–5 % for bread. The present estimates of 1⋅4 g per serving and 11 % for the contribution of miso soup were similar to those in these previous reports. The 3 % contribution of pickles was similar to a more recent study. However, the 3⋅2 g per serving of ramen noodle was smaller and the contribution of bread (10 %) larger than those values previously reported. While assessing the agreement between values estimated in different populations by different methods is difficult, the estimated salt intake was comparable to that described in previous reports.

The above two studies also analysed the sources of salt in the entire Japanese diet. One study^(25)^ showed that processed foods were the major source of salt. The processed foods they referred to included seasonings used discretionally at home, but their contribution in home cooking was not indicated. Another study^(9)^ assessed discretional salt intake and showed that seasonings and ingredients used at home accounted for more than half of total salt consumption. While these results revealed that seasonings were the largest source of salt, information was lacking on the major usage of these seasonings (i.e. how much salt was used for which meals), which is essential for providing education on how to reduce salt content in home cooking.

In the present study, we conducted a diet-wide, dish-based analysis of salt intake. These findings, along with the correlations between the frequency of grain dishes and other dishes, suggest several practical strategies for improving the diet of the Japanese population. First, to promote both salt reduction and dietary balance, plain rice should be chosen as the grain dish, accompanied by salt-reduced fish/meat and vegetable dishes. This strategy is expected to be effective because fish/meat and vegetable dishes are the largest salt sources and because plain rice is the most frequent grain dish. In addition, as recipes for salt-reduced dishes need to be collected from books and websites to promote this dietary shift, public activities that ensure the quality of such information will be required^(26,27)^. Second, noodle dishes should be restricted in frequency, as they tended to impair dietary balance by reducing the consumption of fish/meat and vegetable dishes and because they are heavily seasoned in order to be eaten alone, leaving little room for salt reduction. Third, dairy products to supply calcium, which is generally low in the Japanese diet^(28)^, would be increased when choosing bread as a grain dish. However, noting that bread contains a substantial amount of salt, its consumption alongside other high-salt dishes should be avoided. Fourth, since there are no grain dishes that increase fruit intake, ensuring that the regular consumption of fruits is necessary.

Ideally, practical salt-reducing guidance would indicate dietary goals covering the entirety of the daily diet. Based on the dietary patterns and salt intake obtained in the present study, such a comprehensive guide may be constructed as illustrated in Supplementary Table S2 and Fig. S3. A dietary guide based on daily eating patterns would be useful, as such a guide can be used intuitively without any education required, so not only the people who cook meals but also those who just eat them can understand; furthermore, such an approach closely reflects the actual eating habits of many residents.

Several limitations of the present study warrant mention. First, the study population was a small group in one region of Japan. Although regional differences in dietary habits exist^(29)^, we used dish categories that would allow the appropriate classification of any plate. Furthermore, while the participants were included in a clinical trial and may thus have adopted slightly different dietary patterns from the norm, the total salt intake of the participants was close to the average of the Japanese population; such discrepancies should therefore not critically hamper the estimation of the amount of salt in each dish and its utilisation to guide home cooking. Second, we used a self-monitoring device to estimate the dietary salt intake. Salt excretion assessed with this device has been shown to agree with that estimated by the 24-h urine collection method^(18)^, and in the present study, the mean values were consistent with those estimated from a spot urine sample, suggesting that the systematic error would only be slight. Third, we did not include information on the amount of food eaten in the survey, largely because we focused on reducing responders’ burden and improving the simplicity of deliverables without the use of portion size. This approach is in line with a recent statement concerning the appropriateness of energy intake as assessed by the change in body weight rather than by summing up the amount of food^(5)^.

In conclusion, using the findings from a dish-based dietary survey and evaluation of the salt intake of Japanese community residents, we estimated the salt intake by dish category and the effect of grain dish choices on the intake of other dishes. These results support the possibility of deriving a comprehensive dietary guide that both reduces salt intake and ensures dietary balance in the Japanese diet. Further studies to validate the results in other regions and to apply such a guide in practice are warranted.

## Supplementary material

For supplementary material accompanying this paper visit https://doi.org/10.1017/jns.2021.88. click here to view supplementary material
