# Supplementary material for: Salt intake per dish in the Japanese diet: a clue to help establish dietary goals at home
Source: J Nutr Sci. 2021 Dec 15;10:e107. doi: 10.1017/jns.2021.88 (PMC8727723; doi:10.1017/jns.2021.88)
Supplement: Supplementary file 1 [file S2048679021000884sup001.pdf]

Supplemental Table 1. Representative foods included in each food category

Name of dish category	Main food items (example)
Grain dishes (as staple dishes)	
Plain rice	Plain rice "gohan"
Seasoned rice	Mixed rice "takikomigohan" , fried rice, rice ball, pilaf
Bread	Plain bread, sweet bread, croissant, bagel
Ramen noodles	Soy sauce ramen, miso ramen, wonton noodle
Other noodles	Udon, soba, spaghetti, rice noodles "bihun"
Other grain	Mochi, Japanese pizza, pancake, meat bun "nikuman"
Other dishes	
Fish/meat dish	Grilled fish "yakisakana", grilled meat "yakiniku", fried chicken, raw fish "sashimi", "natto"
Vegetable dish	Salad, boiled greens, vinegared vegetable/ seaweed, grilled mushroom
Dairy products	Milk, yogurt, cheese
Fruits	Orange, apple, banana, strawberry
Optional dishes	
Miso soup	Miso soup, tofu miso soup, miso soup with pork and vegetables
Other soup	Tofu soup, corn soup, potage, minestrone,
Pickles	Pickled radish, takana pickles, boiled in soy sauce "tsukudani"
Mix	
Seasoned rice + fish/meat dish	Curry and rice, beef bowl, omelet rice, tempura on rice, pork cutlet on rice, "nigiri sushi"
Bread + fish/meat dish	Sandwich, hamburger
Fish/meat dish + vegetable dish	"sukiyaki", "syabu-syabu", meat and potatoes "nikujyaga"

Mix: dishes combining a grain dish and other dishes were classified into both categories.

Supplemental Table 2. An example of the recommended frequencies and salt content of each food category to achieve a daily salt intake of 7.0 g (49.0 g/week)

	Average in the study subjects			Recommended values		
Food category	Frequency (times/week)	SI_{dish} (g)	Salt intake (g/week)	Frequency (times/week)	Salt content (g)	Salt intake (g/week)
Plain rice	9.3	0.0	0.0	9.0	0.0	0.0
Seasoned rice	3.7	1.2	4.5	2.0	1.2	2.4
Bread	3.1	1.9	5.9	7.0	1.0	7.0
Ramen noodles	0.6	3.0	1.8	0.5	3.0	1.5
Other noodles	1.5	2.2	3.3	2.0	2.2	4.4
Others	0.7	1.3	0.9	0.5	1.3	0.7
Fish/meat dish	13.5	1.6	21.1	14.0	1.2	16.8
Vegetable dish	12.0	0.9	11.0	14.0	0.6	8.4
Pickles	2.3	0.6	1.4	2.0	0.4	0.8
Miso soup	4.6	1.5	6.9	7.0	1.0	7.0
Other soup	0.8	1.6	1.3			
Fruits	4.6	0.0	0.0	7.0	0.0	0.0
Dairy products	3.0	0.0	0.0	7.0	0.0	0.0
Snack	4.1	-	-	-	-	-
Total			58.1			49.0

The right column show model dietary guidance derived based on based on the mean values in the present study and the following strategies: (i) determining target salt intake (49.0 g/week) and frequencies of necessary dishes (14 fish/meat dishes, 14 vegetable dishes, 7 daily products, and 7 fruits per week), (ii) adjusting the frequency of each dish to achieve the target value, (iii) selectively reducing the intake of salt from fish/meat and vegetable dishes (1.2 and 0.6 g/dish, respectively), which are frequently eaten and account for a large proportion of salt intake, and (iv) limiting the frequencies of dishes in which further reducing salt is difficult, such as noodles and soups.

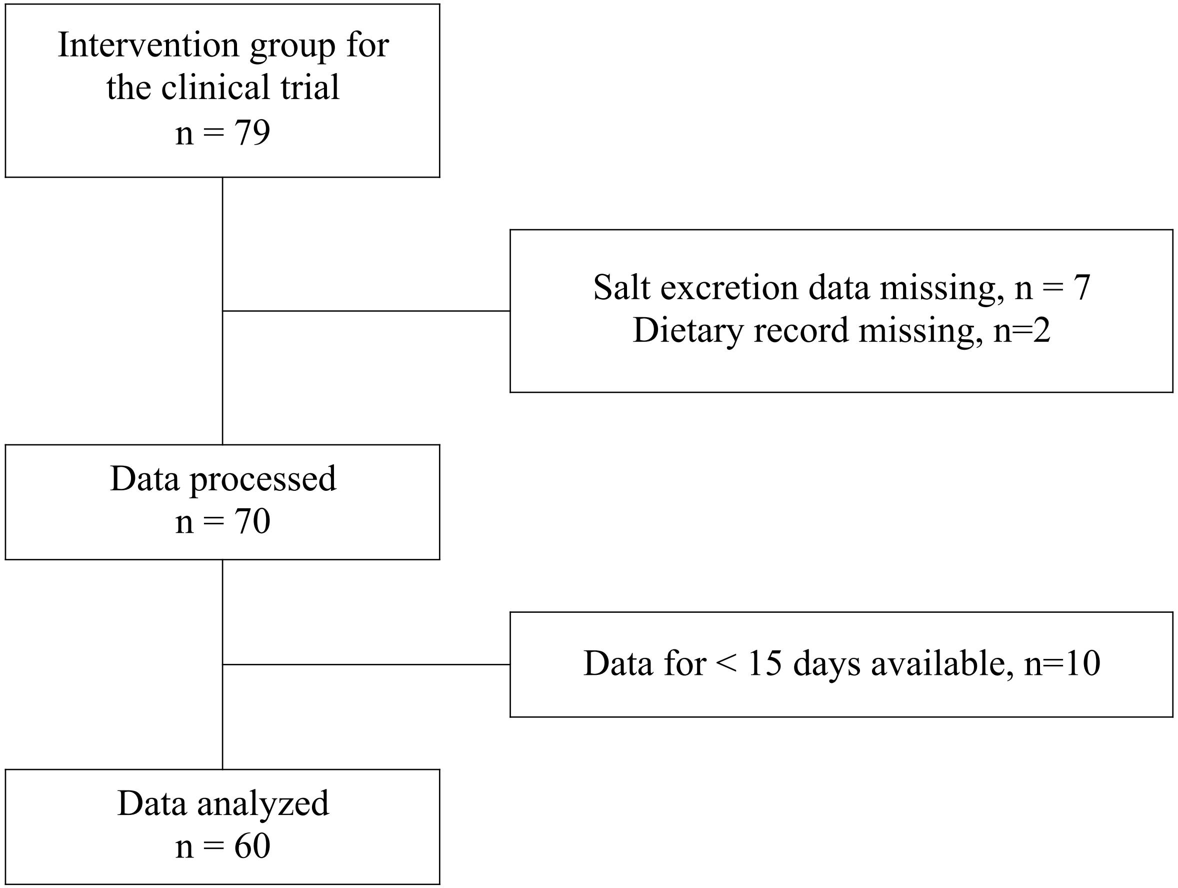

Supplemental Figure 1. Flowchart for selection of study subjects

Please enter briefly the contents of breakfast, lunch, dinner, and snack

Date	Content of meal (briefly)			
	Breakfirst	Lunch	Dinner	between meals
1/1 (Sun)	Rice, miso-soup, natto, apple	Ramen noodles	Rice, soup, grilled fish, fried vegetables, pickles	cookies
	**Sample**			
9/25 (Sun)				
9/26 (Mon)				
9/27 (Tue)				
9/28 (Wed)				

Supplemental Figure 2. Questionnaire used for the food survey in the present study.

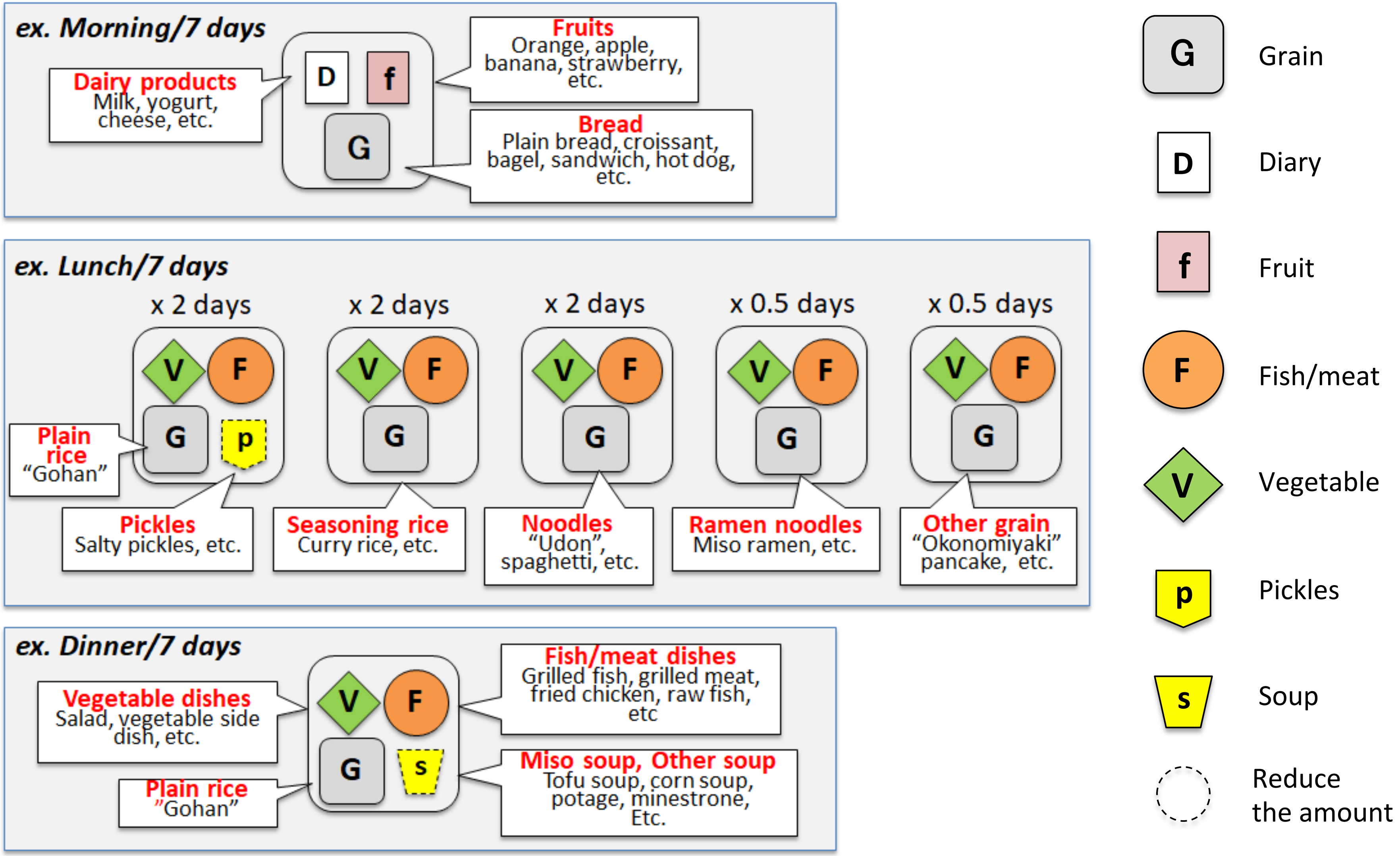

Supplemental Figure 3. A graphical presentation of an example of recommended frequencies of each dish category.
